# Network pharmacology-guided identification of Rabdosia rubescent as a suppressor of laryngeal cancer via MMP9-mediated extracellular matrix remodeling: integrated *in silico* and experimental validation

**DOI:** 10.3389/fphar.2026.1855812

**Published:** 2026-08-12

**Authors:** Xuefeng Wang, Guanying Ren, Yonggang Zhang, Yu Peng, Ruiyao Wang, Yang Li, Jingyu Wang, Sicong Jia, Jingyun Li, Lei Song, Yan Shi

**Affiliations:** 1 Department of Radiation Oncology, Affiliated Hospital of Hebei University, Baoding, China; 2 Hebei Key Laboratory of Cancer Radiotherapy and Chemotherapy, Department of Medical Oncology, Affiliated Hospital of Hebei University, Baoding, China; 3 Department of Head and Neck Surgery, Affiliated Hospital of Hebei University, Baoding, China; 4 Department of Thoracic Surgery, Affiliated Hospital of Hebei University, Baoding, China; 5 Department of Integrated Chinese and Western Medicine, Affiliated Hospital of Hebei University, Baoding, China

**Keywords:** experimental validation, laryngeal cancer, MMP9, molecular docking, molecular dynamics, network pharmacology

## Abstract

**Background:**

Laryngeal cancer, particularly laryngeal squamous cell carcinoma (LSCC), poses a significant threat to global health, especially within Asian populations. While *Rabdosia Rubescens* is known for its antitumor properties, the specific bioactive compounds and mechanisms underlying its activity against laryngeal carcinoma remain to be fully elucidated.

**Methods:**

This study employed an integrated approach combining network pharmacology, molecular docking, and 500 ns molecular dynamics simulations to screen bioactive compounds from *Rabdosia Rubescens*. Key therapeutic targets were identified and validated using transcriptomic data from The Cancer Genome Atlas (TCGA). The most promising candidate was subsequently validated *in vitro* using cytotoxicity assays (TU177 and RK33 cell lines) and wound healing migration assays, alongside MMP9 knockdown models to confirm mechanistic specificity.

**Results:**

Network pharmacology identified MMP9 as a critical therapeutic target, which was found to be significantly overexpressed in LSCC tissues. Virtual screening and MD simulations identified Cirsiliol as the most potent inhibitor among the tested phytochemicals, exhibiting a highly favorable binding free energy (ΔG_binding_ = −179.793 kJ/mol) and stable interactions with key residues (ALA189, HIS266, and LEU243). Experimental validation demonstrated that Cirsiliol induced dose-dependent growth inhibition with IC_50_ values of 31.59 μM in TU177 cells and 17.73 μM in RK33 cells. Furthermore, the compound significantly suppressed cell migration, mirroring the effects observed in MMP9-knockdown TU177 cells.

**Conclusion:**

This study establishes Cirsiliol as a potent, specific inhibitor of MMP9 derived from *Rabdosia Rubescens*. By effectively targeting the MMP9 pathway, Cirsiliol suppresses tumor invasiveness and migration, highlighting its potential as a promising lead compound for the treatment of laryngeal cancer.

## 1 Introduction

**FIGURE 1:**
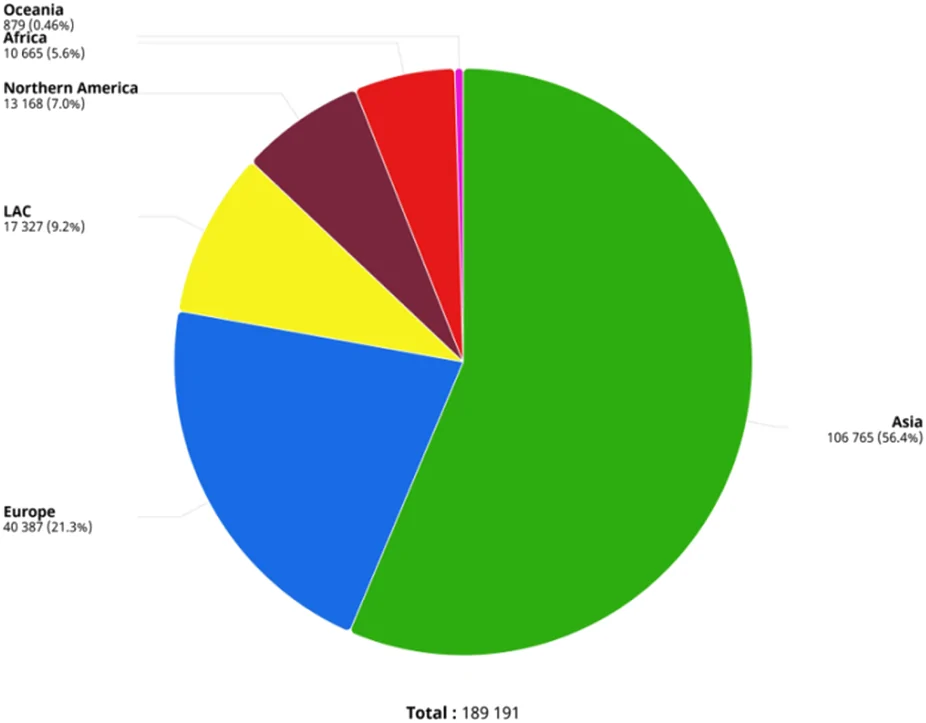
Global cancer incidence and mortality by tumor type. Blue: Estimated global incidence of major cancers. Red: Estimated global cancer mortality by tumor type.

**FIGURE 2:**
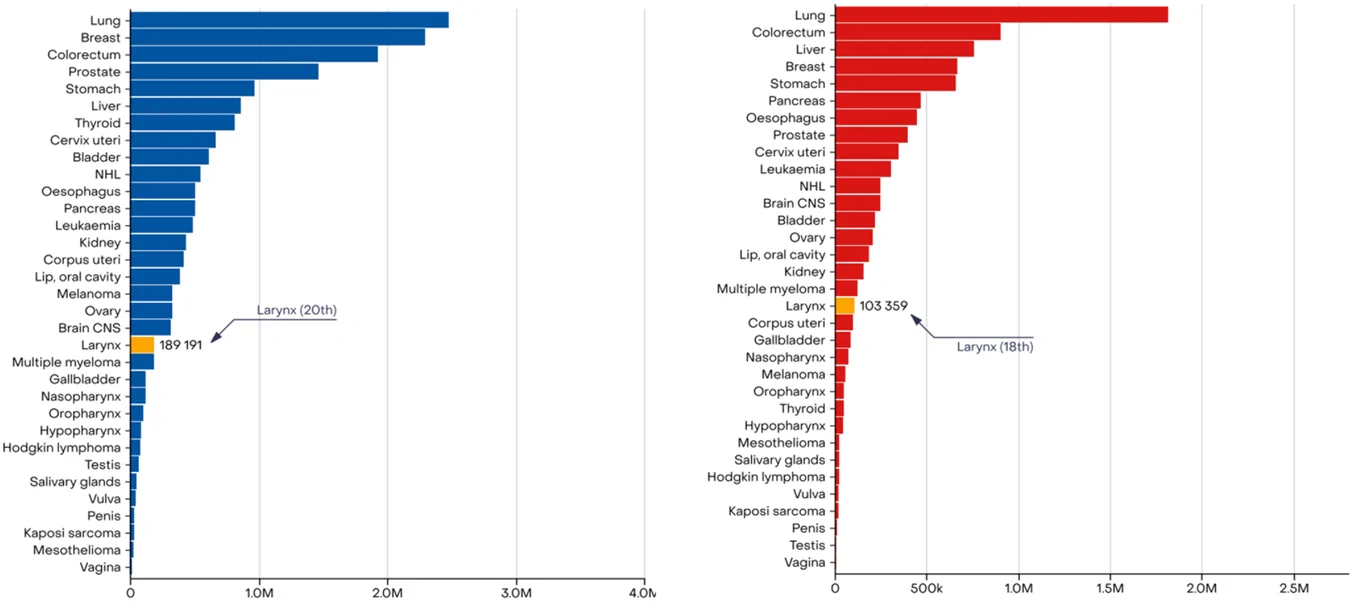
Absolute numbers (both sexes), Incidence of Larynx in 2022–2024.

**FIGURE 3:**
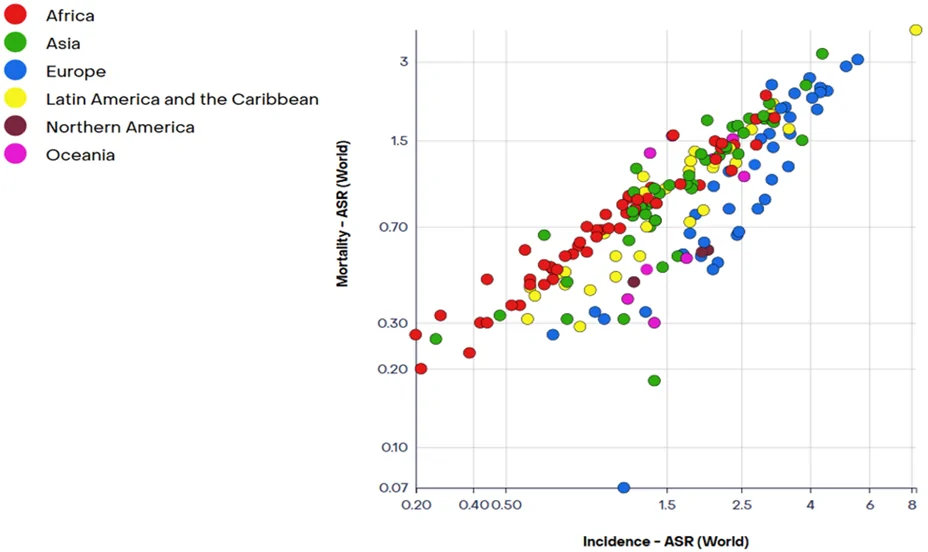
Mortality - ASR (World) vs. Incidence - ASR (World) of Larynx (Both sexes) in 2022–2025.

Laryngeal cancer remains a significant global health burden. According to GLOBOCAN 2022, it accounted for approximately 189,200 new cases and 103,400 deaths worldwide (Figure 1), with the highest incidence and mortality rates observed in Asia and medium Human Development Index countries (Figure 2). Despite advancements in surgical and radiotherapeutic interventions, the prognosis for advanced-stage patients remains poor due to local invasion and lymph node metastasis (Hut et al., 2025). According to “Global Cancer Statistics” for 2022–2024 (https://gco.iarc.who.int/), with an age-standardized incidence rate (ASR) of 1.9 and 189,191 new cases worldwide, laryngeal cancer is the 20th most common type of cancer (Figure 3). Furthermore, conventional therapies often entail severe side effects that compromise patient quality of life. Consequently, there is an urgent need to explore novel, effective, and well-tolerated adjuvant therapeutic agents, particularly those derived from natural products, to improve clinical outcomes and mitigate the side effects associated with standard laryngeal cancer treatments (Hut et al., 2025; Lechien et al., 2022; Qi et al., 2023; Ali Abdalla et al., 2022; Xiao et al., 2025; Zhou et al., 2025).

*Rabdosia Rubescens (Hemsl.) H. Hara,* a prominent herb in Traditional Chinese Medicine (TCM), has been historically utilized for treating upper respiratory and digestive tract disorders, including hoarseness, sore throat, and “Yege syndrome” (esophagitis and esophageal cancer) (Gao et al., 2025). Modern pharmacological studies have identified oridonin, an *ent*-kaurane diterpenoid, as its primary bioactive constituent, exhibiting potent anti-tumor, anti-inflammatory, and anti-angiogenic properties. Preclinical evidence demonstrates that *R. Rubescens* extracts and their active components can induce apoptosis and inhibit proliferation in various human cancer cell lines. Although several clinical formulations of *R. Rubescens* are currently used as adjuvant cancer therapies, systematic investigations elucidating its specific pharmacological mechanisms and therapeutic potential against laryngeal cancer remain scarce (Ali et al., 2024). As adjuvant laryngeal cancer therapy, several formulations, including tablets, syrups, and capsules, have been developed for clinical use. There are still few systematic clinical trials on its anticancer activity, despite its demonstrated safety and pharmacological potential (Oesterle et al., 2019; Ni et al., 2024).

In recent years, with the rapid advancement of systems biology, big data analytics (Li and Zhang, 2023), and artificial intelligence (Li et al., 2025), network pharmacology (Panossian, 2025) has emerged as an innovative research paradigm with significant potential in elucidating drug action mechanisms. Unlike the traditional “one drug-one target” model, network pharmacology emphasizes multi-target and multi-pathway regulation, which is highly consistent with the pharmacological characteristics of traditional Chinese medicine (TCM), namely “multi-component, multi-target, and holistic synergy” (Li et al., 2020). The complex, multi-component nature of TCM poses a challenge for conventional “one drug-one target” pharmacological evaluations. To bridge this gap, network pharmacology has emerged as a robust paradigm for mapping the “multi-component, multi-target, and multi-pathway” synergistic mechanisms of herbal medicines (Zahra et al., 2023; Zhai et al., 2025; Shao et al., 2025; Liu et al., 2025). When integrated with molecular docking and molecular dynamics simulations, this approach not only predicts potential bioactive compounds and therapeutic targets but also validates the binding affinity, spatial conformation, and thermodynamic stability of ligand-receptor interactions at the atomic level (Che and Zhang, 2025; Wang et al., 2023). Despite the proven efficacy of this integrative computational strategy in drug discovery, the underlying molecular network and dynamic interaction mechanisms of *R. Rubescens* in the treatment of laryngeal cancer have not been systematically characterized.

Therefore, this study aims to elucidate the multi-target pharmacological mechanisms of *R. Rubescens* against laryngeal cancer. By integrating network pharmacology, molecular docking, and MD simulations, we sought to identify the core bioactive constituents, key therapeutic targets, and critical signaling pathways involved. Furthermore, these computational predictions were validated through *in vitro* biological activity assays. Ultimately, this research provides a systematic, evidence-based framework to support the clinical application of *R. Rubescens* as an adjuvant therapy for laryngeal cancer and facilitates the modernization of traditional empirical medicine.

## 2 Materials and methods

First, representative bioactive compounds were collected by mining literature, based on their previously reported *in vitro* and *in vivo* pharmacological activities. Subsequently, the potential targets of these compounds were predicted, and laryngeal cancer-related therapeutic targets were retrieved from the GeneCards, OMIM, TTD, and PharmGKB databases. By intersecting the compound targets with the disease targets, the overlapping therapeutic targets were identified. Gene Ontology (GO) functional and KEGG pathway enrichment analyses were then performed on these overlapping targets to explore the involved biological functions and signaling mechanisms. Furthermore, protein-protein interaction (PPI) network was constructed using Cytoscape to identify the key hub genes. Finally, molecular docking and MD simulations were conducted on the primary bioactive compounds to evaluate their binding affinity and structural stability with the core targets. Additionally, *in vitro* bioassays were integrated to validate their therapeutic effects.

### 2.1 Identification of laryngeal cancer-associated targets and overlap analysis

In this study, we systematically collected the major bioactive constituents of *R. Rubescens* through an extensive literature review and targeted database retrieval. The screening process focused on compounds with clearly documented antitumor activities supported by both *in vitro* and *in vivo* studies, as well as constituents with reported or potential clinical relevance. These curated compounds served as the basis for subsequent target identification and network pharmacology analyses. The term “Laryngeal cancer” was used to extract genes linked to Laryngeal cancer from the GeneCards database (https://www.genecards.org/). Only genes with a relevance score higher than 1.0 were chosen to guarantee a strong relationship with the illness.

Based on previously identified chemical profiles of *R. Rubescens* (Gao et al., 2025; Chen et al., 2022), their potential therapeutic targets were predicted using the SwissTargetPrediction database (http://www.swisstargetprediction.ch/), applying a probability threshold of >50% (Daina et al., 2019). A total of 102 potential targets were identified for the 50 active compounds of *R*. *Rubescens* via target prediction. Venn diagram analysis was used to identify 53 overlapping genes, which were then thought to be possible targets for treatment. The links between bioactive chemicals, their targets, and *R*. *Rubescens* were then visualized by building an ingredient–target–disease interaction network using cytoscape.

### 2.2 Genetic functional enrichment analysis

Metascape (Zhou et al., 2019) (https://metascape.org/) was used to conduct KEGG pathway enrichment analysis in order to look into the possible biological pathways linked to the 53 overlapping genes between targets of laryngeal cancer and predicted targets of *R*. *Rubescens* components. Pathways with a p-value < 0.01, minimum count of 3, and enrichment factor > 1.5 were deemed significantly enriched. The analysis was performed using default parameters. The findings indicated a number of signaling pathways and cancer-related pathways that might be involved in *R*. *Rubescens*’s ability to treat laryngeal carcinoma. Bar plots and network diagrams produced automatically by Metascape were used to display the top enriched KEGG pathways, offering insights into the possible mechanisms of action. The pathway-based data were integrated and visualized using Pathview (Luo and Brouwer, 2013). For additional interpretation and incorporation into later investigations, all enrichment results were downloaded.

### 2.3 Molecular docking preparation

Molecular docking was performed between the predicted target proteins in the main pathways and ingredients. The 3D structures of target proteins were obtained from the Protein Data Bank (PDB) (Table 1), while the structures of the small molecules were generated and minimized using RDKit via Python. PyRx 0.8 was used to prepare the proteins and small molecules, including the merging of non-polar hydrogens, the addition of polar hydrogens, and the conversion to the PDBQT format for docking. Molecular docking using AutoDock Vina 1.2.3 was used to find molecules with a favorable binding potential toward the chosen target proteins (Trott and Olson, 2010). In order to balance docking accuracy and computational performance, the exhaustiveness value was always set to 10. The search space was established using the known functional domains or ligand-binding pockets of the proteins, and all docking simulations were carried out inside the active site region of each target. The best binding pose of each ligand from the high exhaustiveness docking stage was analyzed using Discovery Studio 4.5 to characterize its interactions with the kinase pocket. This study aimed to identify potential candidates that occupy binding sites.

**TABLE 1:** PDB ID of receptor.

Targets	PDB ID
AKT1	1h10
IGF1R	1p4o
MET	4r1v
PIK3R1	5aul
PTK2	6yoj
PIM1	8r0q
MMP1	1hfc
DAPK1	4pf4
ALK	4z55
MMP9	6esm
EGFR	8a27

### 2.4 Molecular dynamics simulation

The top-ranked protein–ligand complexes from molecular docking results were further subjected to MD simulations to assess the stability of interactions. Ligands and proteins were parameterized using GAFF2 (He et al., 2020) and ff19SB (Tian et al., 2019) force fields, respectively. The complexes will be solvated in a TIP3P (Price and Brooks, 2004) box at a radius of 8 Å and neutralized with Na + or Cl−. Simulations were carried out using GROMACS 2020.6 (Abraham et al., 2015). All systems were initially energy-minimized using the steepest descent algorithm. Subsequently, equilibration was performed under both NVT and NPT ensembles with holonomic constraints applied. This was followed by a 500 ns production run under the NPT ensemble at a constant pressure of 1 bar, maintained using the Parrinello–Rahman method.

### 2.5 Trajectory analysis

Trajectory analysis was conducted using the MDAnalysis (Michaud-Agrawal et al.), while principal component analysis (PCA) will be performed using the bio3d (Grant et al., 2006) and ncdf4 packages. The representative intervals of 1 ns in all systems were identified via the CIC package (Wei et al., 2025) and were subjected to the gmx_MMPBSA package calculation via GROMACS 2020 (Valdes-Tresanco et al., 2021). The first two principal components (PC1 and PC2) were used to create a conformational free energy landscape. Based on PCA-derived clustering, the frame that corresponded to the global minimum on the free energy surface was shown to be the most stable binding conformation. Using proprietary scripts, this minimum-energy structure was taken out of the MD trajectory. PyMOL was used to visualize the ingredients-target complexes matching the binding pose, emphasizing important interaction residues and the ligand’s orientation within the binding pocket. Binding free energies were evaluated over these intervals using the Molecular Mechanics Generalized Born Surface Area (MM-GBSA) method as implemented in the MMPBSA.py module (Miller et al., 2012).

### 2.6 Cell toxicity determination

The human head and neck squamous cell carcinoma cell line TU177 and the RK33 cancer cell line were used to evaluate the cytotoxicity of the tested compounds. Cells were seeded into 96-well plates at an appropriate density and allowed to attach overnight. The following day, cells were treated with the compounds at a series of concentrations: 6.25, 9.375, 12.5, 18.75, 25, 37.5, 50, 75, 100, 150, 200, and 300 μM. Untreated cells served as the negative control. After 24 h of incubation, cell viability was determined using the MTS assay according to the manufacturer’s instructions. Briefly, MTS reagent was added to each well and incubated for an additional period at 37 °C. Absorbance was measured at 490 nm using a microplate reader. Cell inhibition rate was calculated using the following formula:

$$\text{Inhibition rate }\left(\%\right)=\left(1-A_{\text{treated}}/\;A_{\text{control}}\right)\times 100$$

Dose–response curves were generated by nonlinear regression analysis, and the half-maximal inhibitory concentration (IC_50_) values were calculated using GraphPad Prism (v9.6.1).

### 2.7 Wound healing assay

The migratory ability of TU177 cells was evaluated using a wound healing assay. Cells were seeded into 6-well plates and grown to approximately 90%–100% confluence. A straight scratch was created across the cell monolayer using a sterile 200-μL pipette tip. Detached cells were removed by gently washing with phosphate-buffered saline (PBS).

Cells were then incubated with either vehicle control (CTL) or the compounds at a final concentration of 20 μM. Representative images of the wound area were captured at 0, 12, 24, and 36 h after scratching using an inverted microscope.

## 3 Results

### 3.1 Identification of common targets and network construction of Rabdosia Rubescens against laryngeal cancer

Initially, thirty representative bioactive published structures were extracted within the last 5 years on the pharmacological potential of *R*. *Rubescens*, with an emphasis on antineoplastic constituents. These structurally diverse metabolites-predominantly steroidal and terpenoid derivatives-were curated and assembled into the preliminary candidate compound library for subsequent analysis.

**FIGURE 4:**
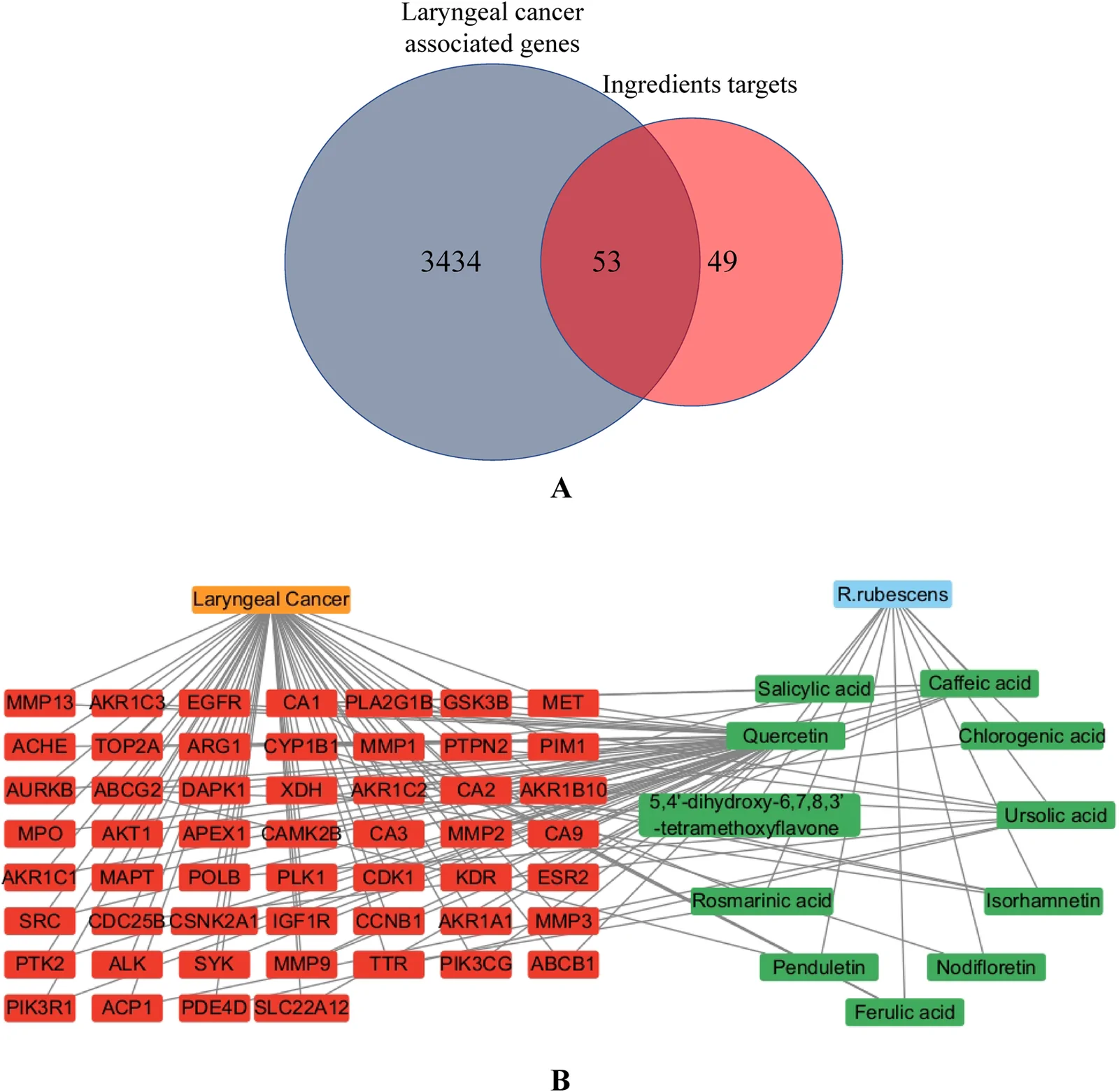
Overlap analysis and compound–target network of R. *Rubescens* against laryngeal cancer. **(A)** Venn diagram showing the overlap between 102 predicted and 3487 genes linked to laryngeal cancer. 53 similar targets were found in the investigation. **(B)** Traditional Chinese Medicine-active components-target (H–C–T) network of Rabdosia Rubescens (blue node) acting on laryngeal cancer (orange node), 11 chemical constituents (green nodes) and 53 targets (red nodes).

The possible targets of *R*. *Rubescens*’ chemical constituents were predicted using the SwissTargetPrediction database. In total, 102 potential targets were identified for the 50 compounds chosen. Table 1 summarizes all targeted proteins included in this study, along with the structural characteristics and related information of the screened bioactive molecules. The GeneCards database was searched for genes associated with laryngeal cancer using the phrase “Laryngeal cancer,” yielding 3,487 results. After merging the two target sets were intersected to find overlapping genes. Venn diagram analysis (http://bioinformatics.psb.ugent.be/webtools/Venn/) identified 53 common targets (Figure 4A) of *R*. *Rubescens* that could be used as therapeutic targets for laryngeal cancer. Next, using Cytoscape 3.10.3 (Figure 4B), a network was built by combining the 53 common targets, *R*. *Rubescens*, and its active chemicals. The network included 53 common targets, 11 active molecules, and *R*. *Rubescens*. The main bioactive components influencing the therapeutic impact against laryngeal cancer may be represented as key active compounds with higher degree values.

### 3.2 Identification of key oncogenic pathways and targets of Rabdosia Rubescens and PPI network analysis

**FIGURE 5:**
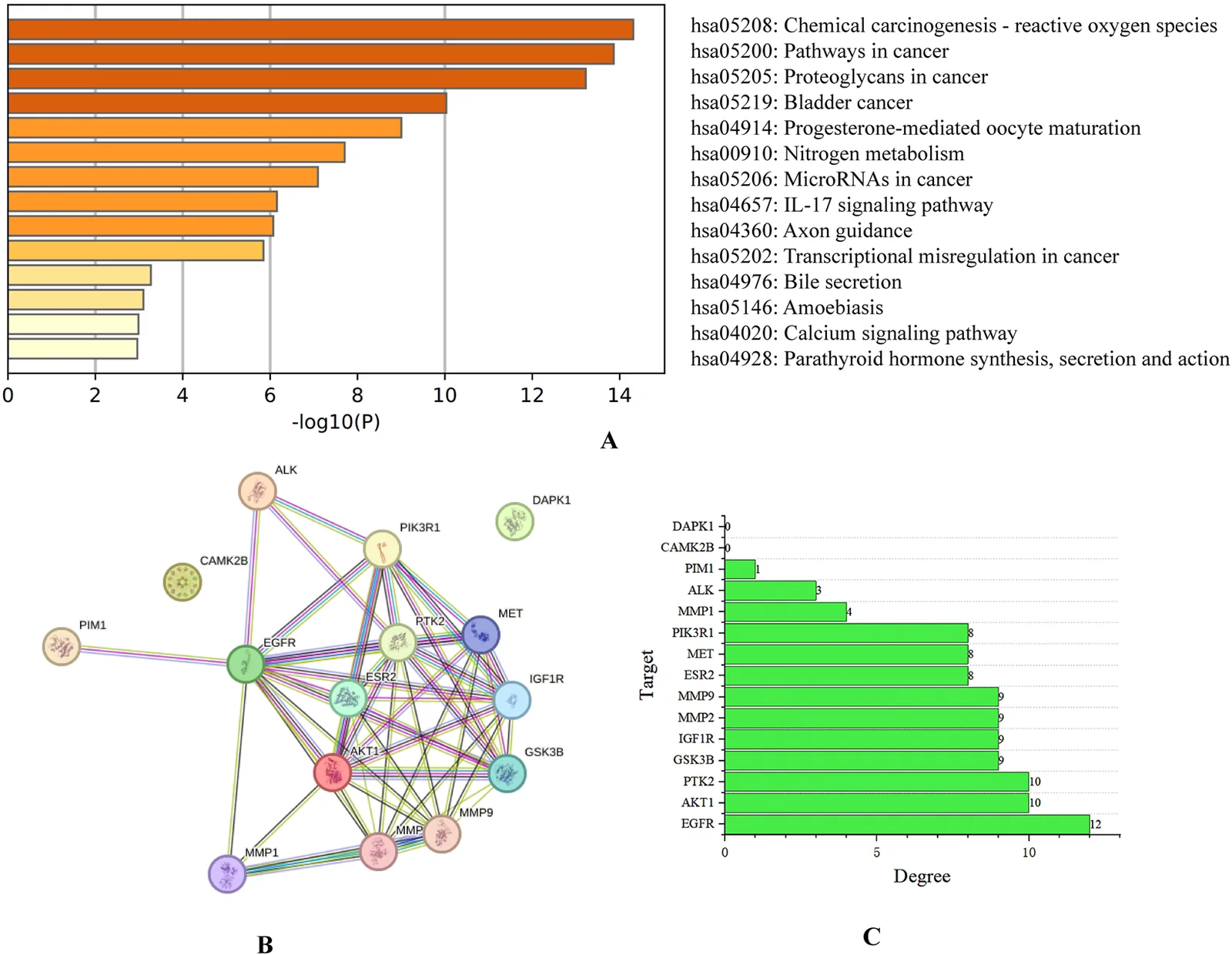
KEGG pathway enrichment and PPI network analysis of common targets. **(A)** KEGG pathway enrichment analysis of the 53 common targets using the Metascape platform, showing the top 14 significantly enriched pathways. **(B)** Protein-Protein Interaction (PPI) Network Diagram (The PPI network of 15 potential targets). **(C)** Bar chart of node degree values in the PPI network. The horizontal axis shows node degree values (0–12), the vertical axis counts proteins per degree.

Enrichment analysis of the 53 overlapping targets was performed using the Metascape platform, and the top 14 enriched KEGG pathways are presented in the ranking chart. (Figure 5A). The hsa05200: Pathways in cancer route ranked second in enrichment significance and was chosen for further examination among them because it is a thorough network of important oncogenic and tumor-suppressive signaling cascades linked to several cancers, including laryngeal carcinoma. AKT1, ALK, CAMK2B, DAPK1, EGFR, ESR2, GSK3B, IGF1R, MET, MMP1, MMP2, MMP9, PIM1, PIK3R1, and PTK2 are among the 53 overlapping targets that were notably enriched in this pathway. This suggests that *R*. *Rubescens* may have anti-laryngeal cancer effects by multi-target regulation of these key oncogenic drivers and signaling nodes.

Using the STRING database (https://string-db.org/), the protein–protein interaction (PPI) network of the 15 targets enriched in the hsa05200: Pathways in cancer pathway was built, and the PPI network diagram was produced (Figure 5B). EGFR (Li et al., 2025), AKT1 (Ni et al., 2024), PTK2 (Ni et al., 2024), MMP9 (Oesterle et al., 2019), MMP2 (Oesterle et al., 2019), IGF1R (Oesterle et al., 2019), and GSK3B (Oesterle et al., 2019), the top nodes with the highest degree values, suggest that these proteins may be important targets for *R*. *Rubescens* in the treatment of laryngeal cancer and may play central roles in the interaction network (Figure 5C).

In the PPI analysis, the degree of a node represents the number of edges connected to it. Proteins with higher degrees are more likely to serve as central hubs through which drugs exert their effects. Meanwhile, betweenness centrality describes the frequency with which a node lies on the shortest paths between all other pairs of nodes in the network. Proteins with higher betweenness centrality act as “traffic hubs” for information flow, bridging distinct functional modules. In the present analysis, EGFR, AKT1, PTK2, MMP9, MMP2, and GSK3B all exhibited relatively high degrees, suggesting their potential roles as key targets in mediating the pharmacological effects.

**FIGURE 6:**
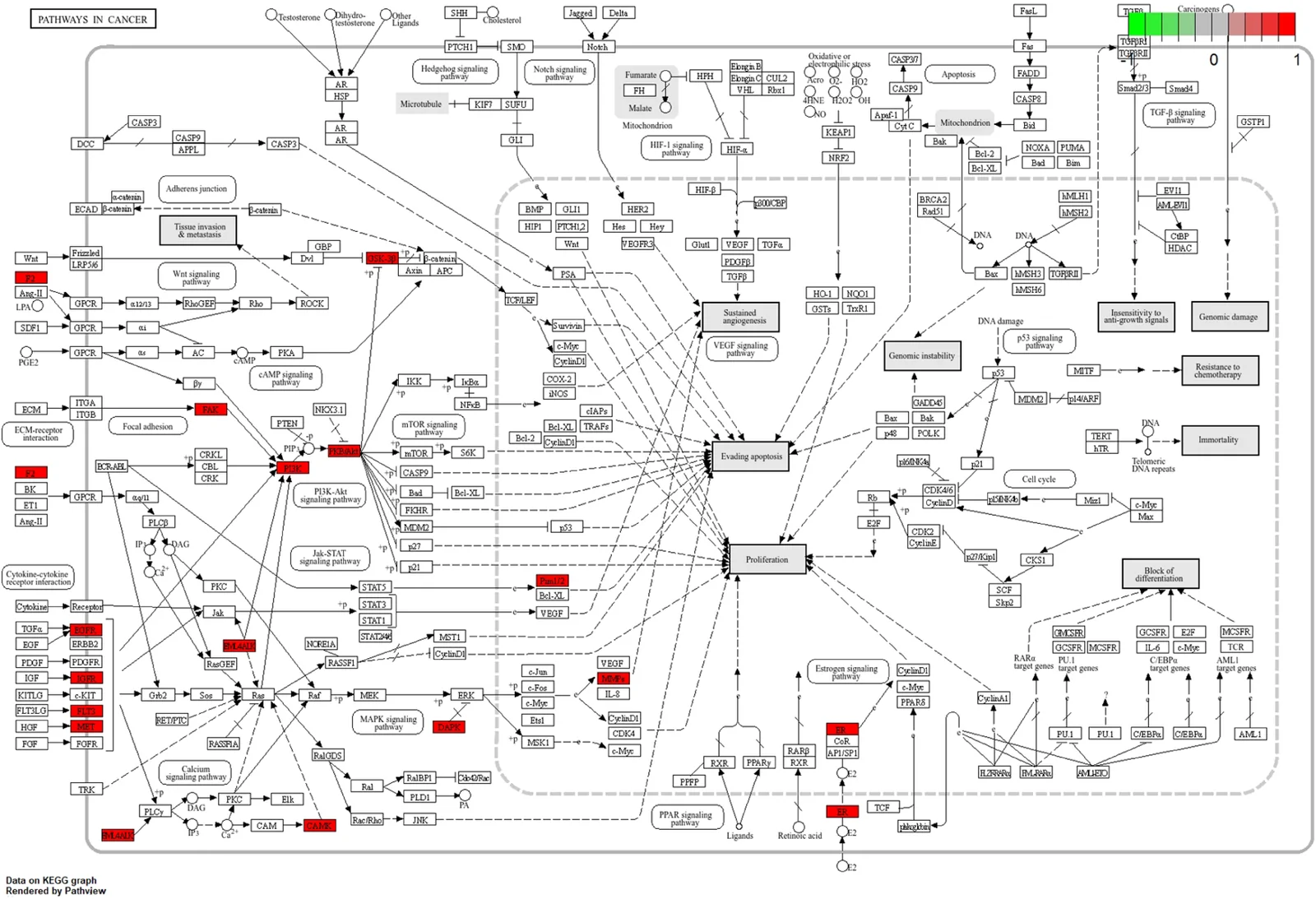
Pathview enrichment results.

According to the Pathview enrichment results, MMP2 and MMP9 were found to be highly activated in signaling pathways linked to cancer (Figure 6), specifically in the modules of invasion and metastasis, focal adhesion, and ECM-receptor interaction.

### 3.3 Docking analysis reveals multi-target binding potential of *Rabdosia Rubescens*

The occurrence, progression, and prognosis of laryngeal cancer have been found to be tightly linked to 11 of the 15 targets enriched in the hsa05200: Pathways in cancer pathway, including AKT1, ALK, DAPK1, EGFR, IGF1R, MET, MMP1, MMP9, PIM1, PIK3R1, and PTK2. Meanwhile, key oncogenic processes like angiogenesis, invasion, metastasis, apoptosis resistance, and abnormal cell proliferation are all impacted by these targets (Hu et al., 2025). As a result, they were chosen for additional molecular docking studies as key therapeutic targets.

**FIGURE 7:**
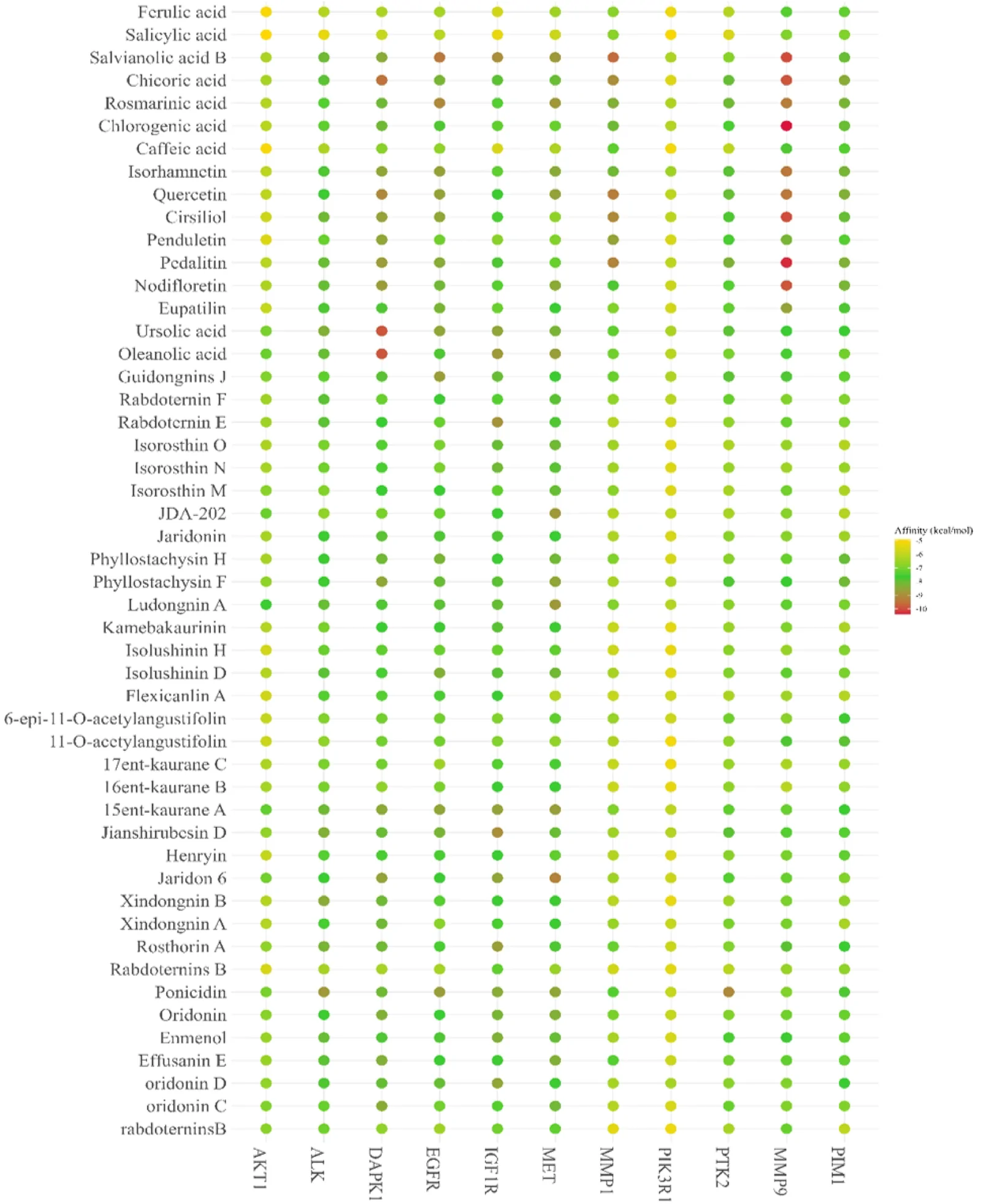
The heatmap indicates molecular docking scores between 11 target proteins linked to laryngeal cancer and 50 bioactive compounds. Docking scores are shown by a color gradient, where green denotes weaker binding and red denotes stronger binding affinities (lower energy values).

To assess their binding potential, the 50 active compounds of *R*. *Rubescens* were docked against the 11 chosen targets. Figure 7 displays the results of the calculation of binding affinity values. The docking results offer a molecular basis for the multi-target therapeutic effects of *R*. *Rubescens* by shedding structural light on the possible interactions between the herb’s bioactive ingredients and key targets linked to laryngeal cancer.

Four compound–target complexes: Pedalitin - MMP9(-10.3 kcal/mol), Cirsiliol–MMP9 (−10.0 kcal/mol), Chlorogenic acid–MMP9 (−10.3 kcal/mol), and Salvianolic acid B–MMP9 (−10.0 kcal/mol), showed binding affinities less than −10 kcal/mol, according to molecular docking studies. According to these findings, the chosen compounds may have a particularly high affinity for MMP-9, a target that is strongly linked to tumor invasion and metastasis in laryngeal cancer.

**FIGURE 8:**
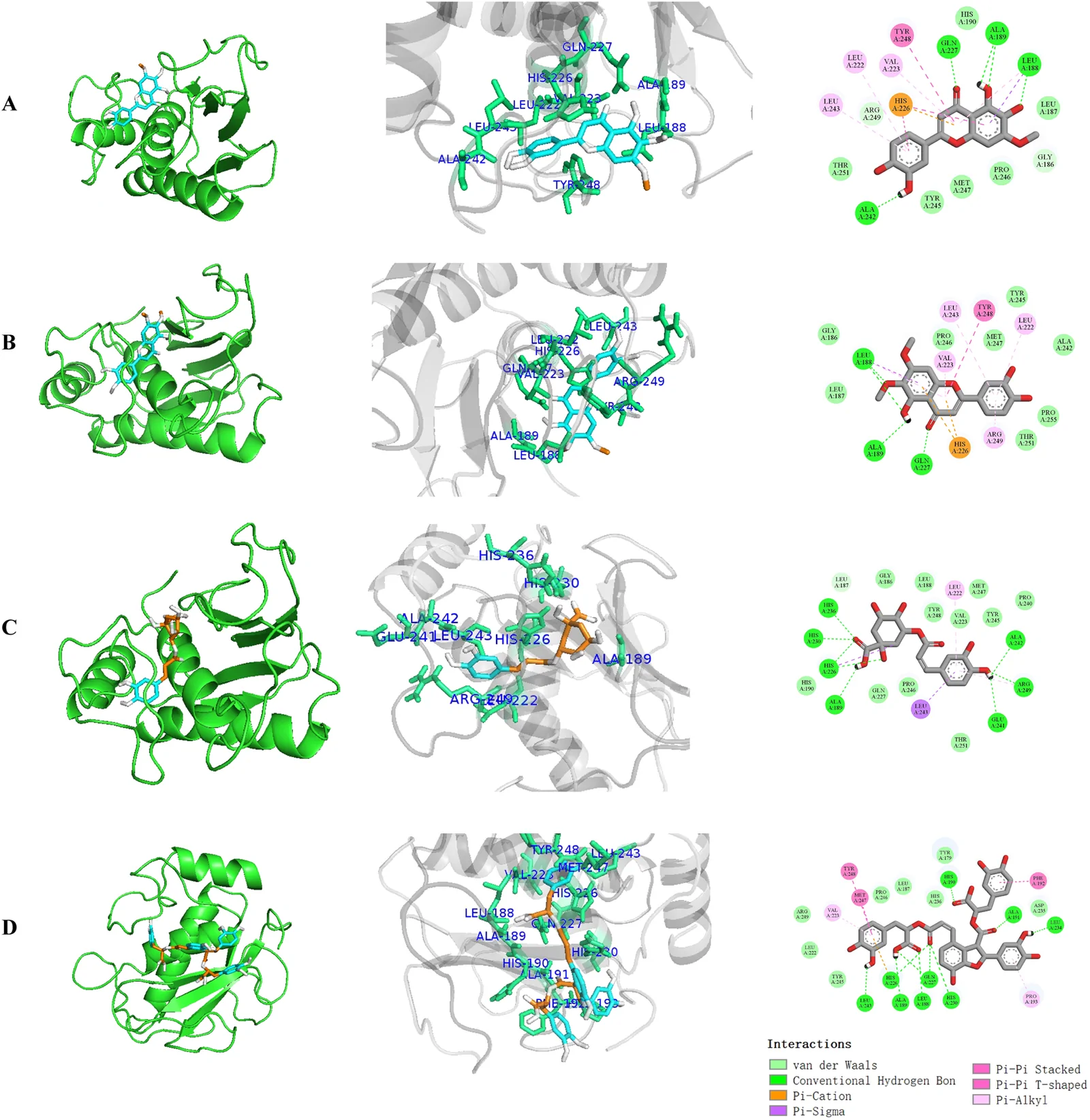
Docking conformation analysis of high-affinity complexes between Rabdosia Rubescens active compounds and MMP9. Each panel shows the ligand position in the full protein (left), the 3D binding mode with interacting residues (middle), and the 2D ligand–protein interaction diagram (right). **(A)** Pedalitin–MMP9, **(B)** Cirsiliol–MMP9, **(C)** Chlorogenic acid–MMP9, and **(D)** Salvianolic acid B–MMP9.

The docking conformations of the four complexes were thoroughly examined in order to learn more about the binding mechanisms (Figures 8A–D). The spatial location of the ligand within the overall protein structure, a 3D representation of the ligand interacting with important amino acid residues, and a 2D ligand–protein interaction diagram showing hydrogen bonds, hydrophobic contacts, and other pertinent interactions were all analyzed for each complex. The structural evidence for these chemicals’ possible inhibitory effects on MMP9 activity is provided by these visualizations.

### 3.4 The dynamics stability simulation of compounds with core receptor proteins

**FIGURE 9:**
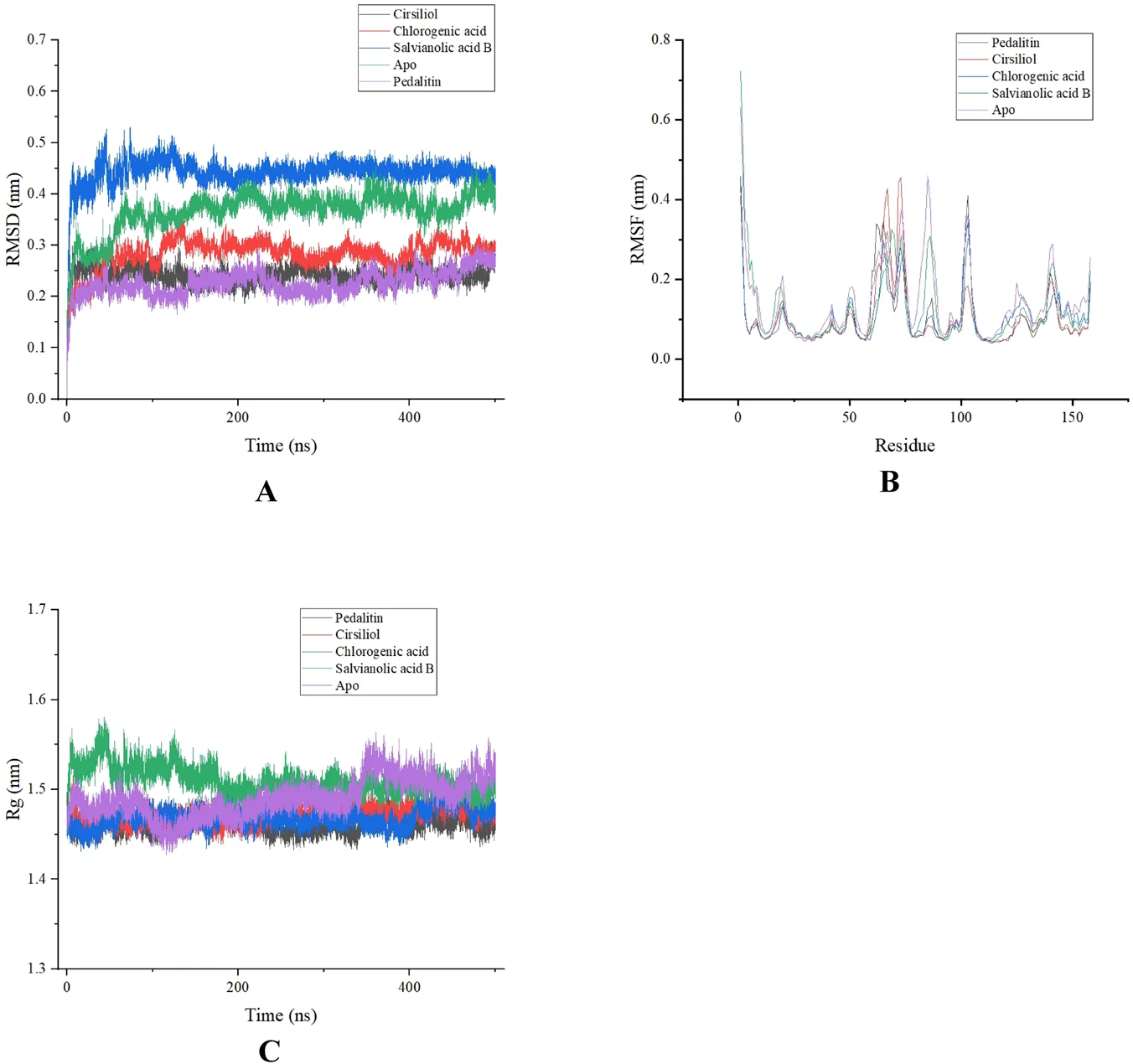
RMSD **(A)**, RMSF **(B)** and Rg **(C)** analysis of complexes between compounds and MMP9.

Molecular dynamics simulation is a crucial tool for investigating the stability and conformational change of the ligand-protein complex following docking. The simulations were performed for 500 ns on 5 selected systems (including 1 Apo system), including complexes of Pedalitin, Cirsiliol, Chlorogenic acid and Salvianolic acid B with MMP9, as well as the apo (ligand-free) form, to further assess the binding stability and structural behavior of the chosen small-molecule–protein complexes. To evaluate structural stability and flexibility, important structural metrics such as the radius of gyration (Rg), root mean square deviation (RMSD), and root mean square fluctuation (RMSF) were examined (Figure 9).

Compared to the apo form, the RMSD graphs suggest that most ligand-bound complexes achieved equilibrium early in the simulation and stayed stable throughout. RMSF explores shed light on oscillations at the residue level. In contrast to the apo form, ligand interaction for most complexes led to less flexibility in the active or binding site regions, indicating that the ligand assisted in stabilizing important functional domains of the target proteins. The protein complexes’ structural integrity and compactness were further validated by the radius of gyration (Rg) profiles. Rg values were comparatively constant across all systems, suggesting that there was no appreciable unfolding or collapse of protein structures throughout the simulation time. These findings collectively demonstrate that the chosen ingredients bind to their respective protein targets to form stable and compact complexes, making them attractive options for additional binding free energy calculations and pharmacological assessment.

**FIGURE 10:**
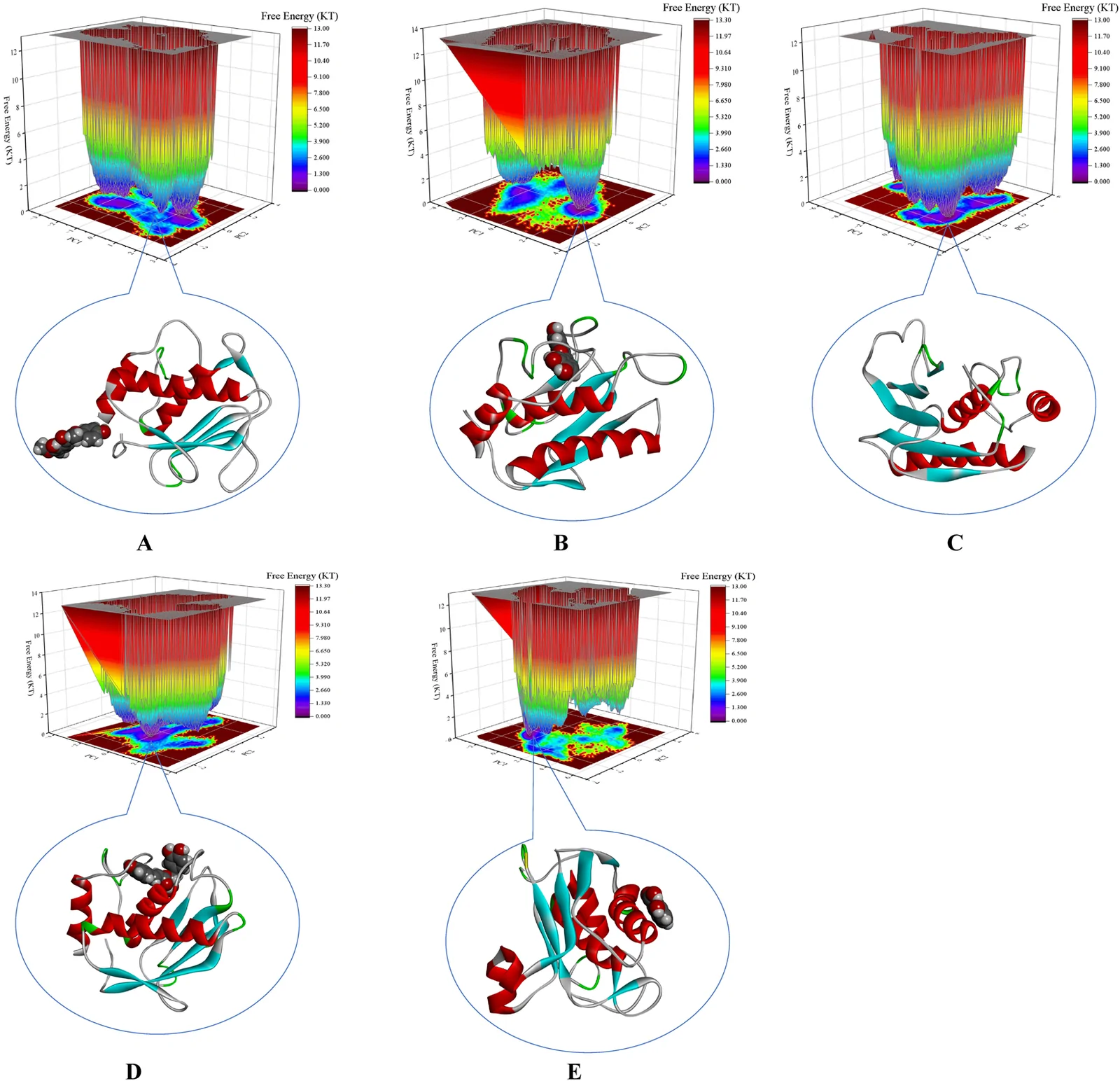
Three-dimensional free energy landscape (3D-FEL) of selected protein–ligand complexes based on PCA. The landscapes were constructed using PC1 and PC2 as the reaction coordinates and free energy (ΔG, in kJ/mol) as the Z-axis. Representative conformations extracted from the global minimum of FELs. **(A)** Pedalitin–MMP9, **(B)** Cirsiliol–MMP9, **(C)** Chlorogenic acid–MMP9, **(D)** Salvianolic acid B–MMP9, and **(E)** MMP9. CPK model represents compounds.

To determine the most stable conformations during the MD trajectories, principal component analysis (PCA) was conducted on the backbone atoms of each protein–ligand complex (Figure 10). The free energy landscape (FEL) was projected onto the first two principal components (PC1 and PC2). The color gradient indicates the free energy (ΔG_bind_, kJ/mol), ranging from high-energy unstable states (red-orange) to low-energy stable basins (blue-purple).

Analysis of the FEL and representative structures revealed significant differences in binding stability. Although Pedalitin and Salvianolic acid B showed favorable docking scores, their global minimum conformations indicated that the ligands had drifted out of the conventional MMP9 binding pocket. This conformational instability suggests a failure to maintain the initial binding mode during the simulation. Conversely, Cirsiliol and Chlorogenic acid exhibited robust stability. Their FELs displayed well-defined low-energy basins, and the extracted representative structures confirmed that these ligands remained securely bound within the active site. These findings emphasize the superior binding persistence of Cirsiliol and Chlorogenic acid compared to the other investigated compounds.

MM-PBSA analysis was performed using 100 frames (1 ns) taken from the global minimum energy basin found by PCA to confirm the thermodynamic stability of certain protein–ligand complexes. The most stable area of the conformational space observed during MD simulations is represented by these frames.

**FIGURE 11:**
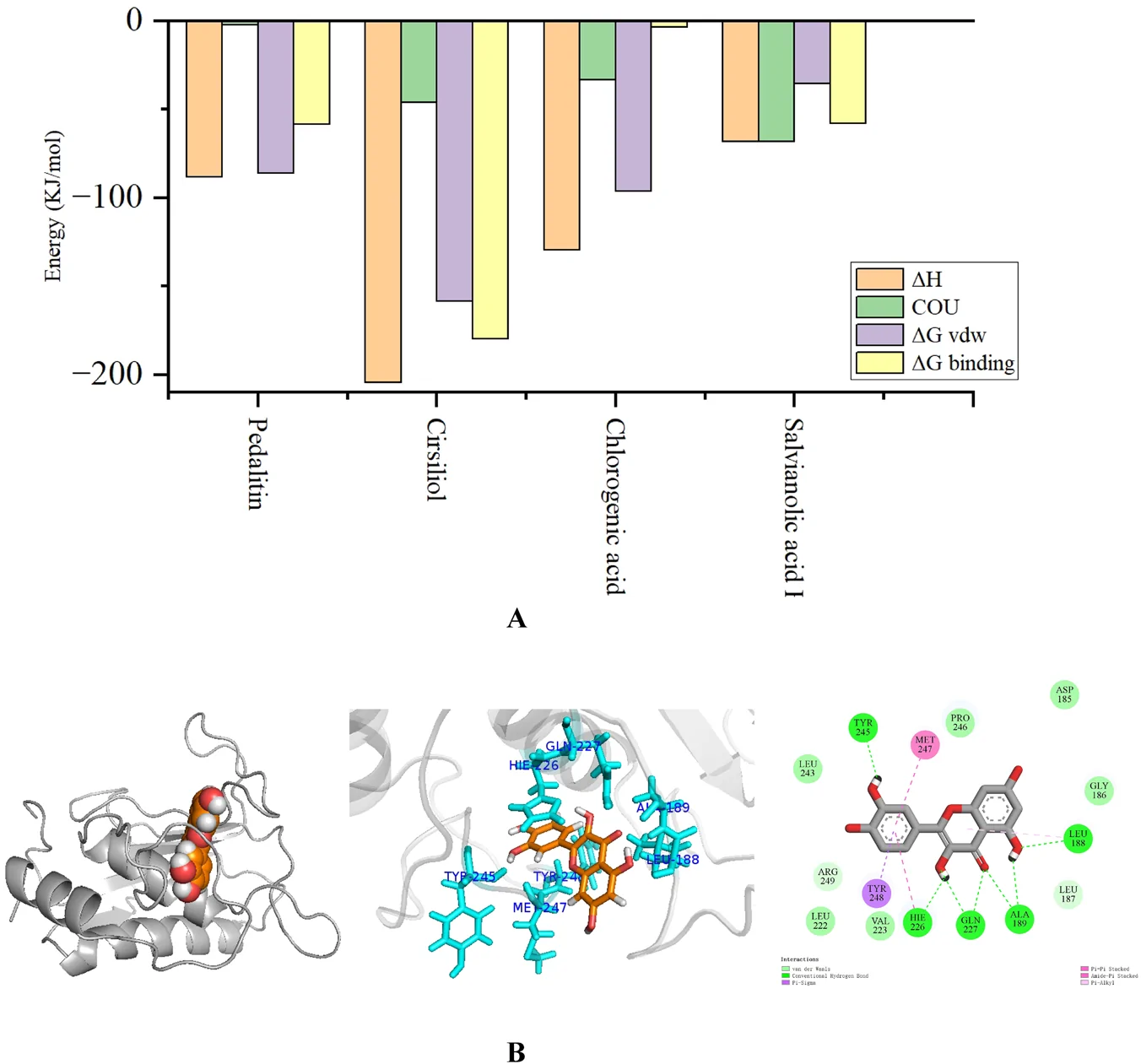
**(A)** Bar plot showing total enthalpy change (ΔH), Coulombic energy (COU), Van der Waals energy (ΔG vdw), and binding free energies (ΔG_binding) for the 4 selected protein–ligand systems, based on 100 frames from the global minimum energy basin identified by PCA. **(B)** Panel shows the ligand position in the full protein (left), 3D binding mode with interacting residues (middle), and 2D ligand–protein interaction diagram (right).

The binding free energies (ΔG_bind_) of the 4 systems that were chosen ranged from −179.793 to −3.36 kJ/mol, as indicated in Figure 11A. The largest binding affinity among them was shown by the MMP9–cirsiliol complex (ΔG_bind_ = −179.793 kJ/mol). This points to extremely stable connections between protein and ligand. Conversely, MMP9-chlorogenic acid (−3.36 kJ/mol) displayed low binding affinity, suggesting weak or unstable interaction that might be brought on by inadequate contacts within the binding site or less-than-ideal ligand orientation. We choose MMP9 – Cirsiliol (<-150 kJ/mol) for further structural analysis.

### 3.5 The cirsiliol suppresses migration of TU177 cells

**FIGURE 12:**
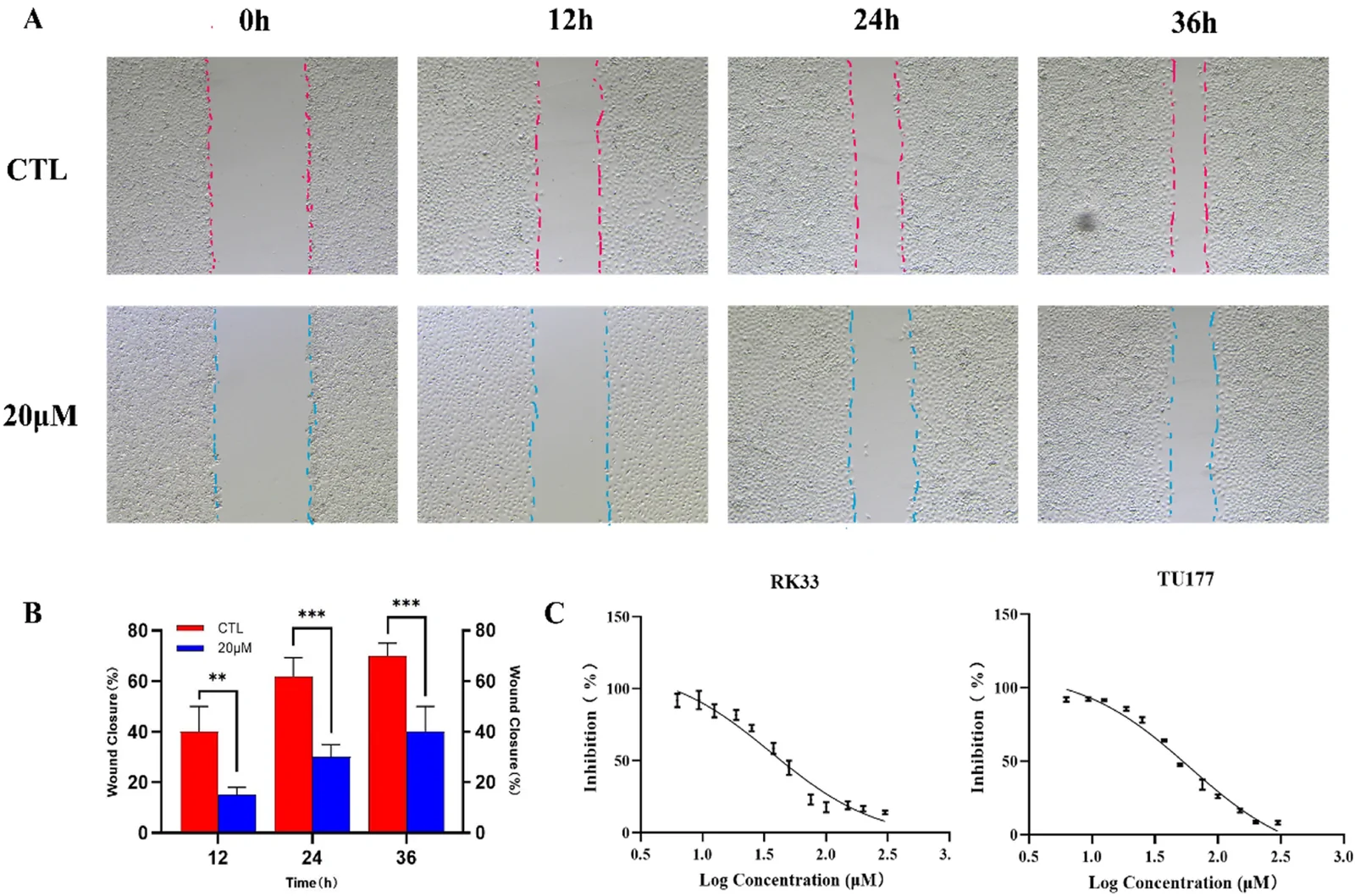
**(A)** The compound inhibits migration of TU177 cells in a wound healing assay. Confluent TU177 cell monolayers were scratched and treated with vehicle control (CTL) or 20 μM of the compound. Representative images of wound closure were captured at 0, 12, 24, and 36 h. **(B)** Statistical analysis of wound healing percentages at 12 h, 24 h, and 36 h under different treatment conditions. All results were from three experiments, and all values were expressed as the mean ± standard deviation. One-way analysis of variance and the Turkey test were used for multiple comparative analyses of differences between the control group and the treatment group. P****<0.0001, p***<0.001, p**<0.01, p*<0.05, ns indicated no significant difference. **(C)** Cytotoxic effects of the compound on TU177 and RK33 cells.

Compared with the CTL, the TU177 cell had clearly migrated into the scratched area and decreased the wound gap significantly at 24 h and nearly completed the wound closure at 36 h. After being treated with 20 M Cirsiliol, the closure of the wound was obviously suppressed, there was still a significant wound area between the two margins at 12, 24, and 36 h than the CTL group (Figure 12A). These data demonstrate that Cirsiliol was able to inhibit the migratory ability of TU177 cells *in vitro*.

### 3.6 Cytotoxic effect of cirsiliol on TU177 and RK33 cells

To determine the anti-cancer efficacy of Cirsiliol, cytotoxicity of Cirsiliol was measured on TU177 cells and RK33 cells by using the MTS assay (Figure 12B). It was observed that both cell lines had a clear dose-dependent killing effect from Cirsiliol treatment (6.25–300 M, 24 h) and the IC_50_ values were determined to be 31.59 M on TU177 cells and 17.73 M on RK33 cells by non-linear regression (Figure 12B), which indicate that the RK33 cell line was more sensitive to Cirsiliol than the TU177 cell line. Above all, these data represent that Cirsiliol inhibit both migration and proliferation activity of human head and neck cancer derived TU177 and RK33 cell lines *in vitro* (Figure 12C).

## 4 Discussion

Laryngeal cancer is a typical head and neck malignancy that poses a significant threat to global human health, particularly among Asian populations. *R. Rubescens*, an important medicinal plant, has been reported to exhibit notable antitumor and other pharmacological activities. In this study, we employed an integrated approach combining network pharmacology, molecular docking, and MD simulations to screen bioactive compounds from *R. Rubescens* with anti-laryngeal carcinoma activity and to elucidate their potential mechanisms. Our *in silico* research provided preliminary evidence for a mechanism centered on the MMP9 pathway and identified cirsiliol as a highly promising inhibitory compound.

**FIGURE 13:**
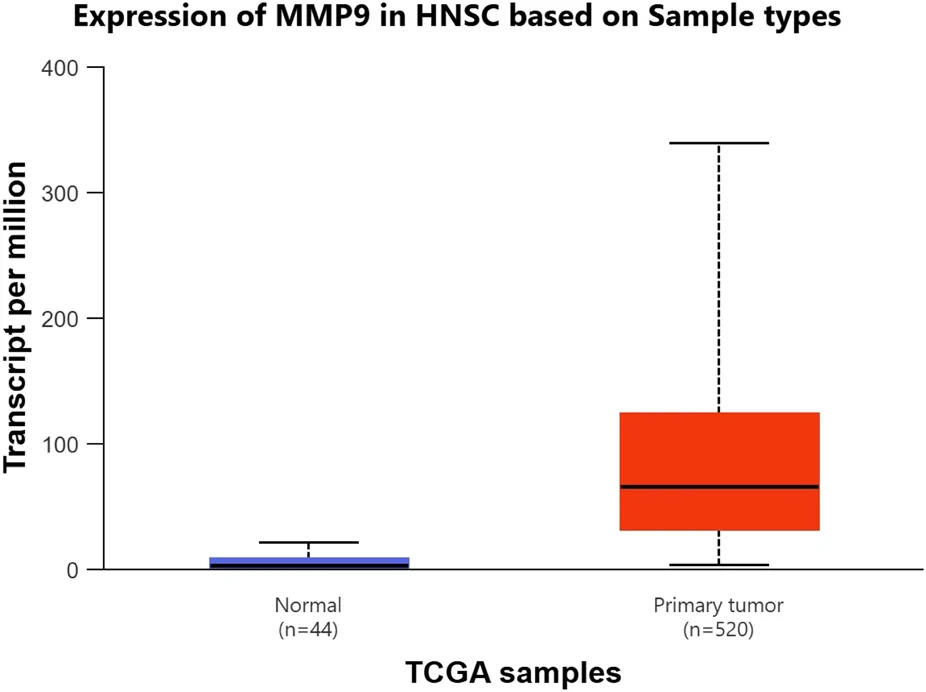
Boxplot of MMP9 expression in HNSCC and normal tissues based on the TCGA dataset.

Our network pharmacology analysis identified MMPs, EGFR, and AKT1 as key therapeutic targets, with their clinical relevance strongly supported by transcriptomic data. AKT1, PTK2, MMP2, MMP9, and GSK3B constitute a tightly interconnected functional network in laryngeal cancer, particularly LSCC. Activation of EGFR stimulates the PI3K-AKT pathway, leading to the activation of AKT1, which in turn inhibits GSK3B and drives downstream processes such as cell survival and Wnt signaling activation. In parallel, EGFR cooperates with PTK2 to regulate tumor cell adhesion and migration, thereby inducing the expression of MMP2 and MMP9, which facilitate extracellular matrix (ECM) degradation and tumor invasion. Collectively, these molecules orchestrate tumor cell proliferation, invasion, and metastasis and are closely associated with poor prognosis in LSCC. Notably, transcriptomic data from the TCGA database (Figure 13) revealed that MMP9 was significantly overexpressed in head and neck squamous cell carcinoma (HNSCC) tumor tissues compared to normal controls. This upregulation aligns with its well-established role in ECM degradation, tumor invasion, and metastasis, underscoring the importance of targeting MMP9 in laryngeal cancer.

MMP9 (matrix metalloproteinase-9), also known as gelatinase B, is a zinc-dependent protease that plays a key “executor” role in the pathogenesis of laryngeal cancer. Compared with adjacent normal tissues, MMP9 is significantly upregulated in laryngeal cancer tissues. Its core pathological function is to degrade the ECM, especially type IV collagen, which is the main structural component of the basement membrane (BM). The BM is a crucial physical barrier separating epithelial cancer cells from the underlying stromal tissue. MMP9 directly promotes the local invasion of cancer cells by breaking down this barrier, paving the way for their subsequent migration through the stroma and intravasation to achieve lymph node and distant metastasis. In addition, MMP9 promotes tumor angiogenesis by remodeling the ECM and releasing matrix-bound growth factors such as VEGF. Therefore, high expression of MMP9 is closely associated with advanced T/N staging, poor differentiation, and poor prognosis, making it a recognized key therapeutic target for inhibiting the invasion and metastasis of laryngeal cancer.

Based on the key target MMP9, our virtual screening ultimately identified four natural compounds with significant binding potential: pedalitin, cirsiliol, chlorogenic acid, and salvianolic acid B. Molecular docking analysis showed that all four phytochemicals exhibited high affinity for MMP9 and consistently bound to key residues (ALA189, HIS266, and LEU243) within the active site (Table 2). This repetitive binding pattern may provide useful pharmacophore clues for the development of specific MMP9 inhibitors. Additionally, a known MMP9 inhibitor, 2-(2-((4′-methoxy-[1,1′-biphenyl]-4-yl) thio) phenyl) pentanedioic acid (Qi et al., 2023), was included as a reference, and its docking conformation also confirmed the importance of the ALA189, HIS266, and LEU243 residues for binding (Figure 11B).

**TABLE 2:** Molecular Docking interactions of individual protein-ligand complexes.

Protein-ligand complex	Hydrogen bond	Hydrophobic interaction
Pedalitin-MMP9	ALA242, ALA189, LEY188, GLN227	TYR248, VAL223, HIS226, LEU222
		ARG249, LEU243
Cirsiliol-MMP9	ALA189, GLN227, LEU188	HIS226, ARG249, LEU222, TYR248
		LEU243
Chlorogenic-MMP9	HIS236, HIS230, HIS226, ALA189	LEU222, LEU243, VAL223, PRO246
	GLU241, ARG249, ALA242	VAL223, TYR245, GLN227
Salvianolic acid-MMP9	HIS190, ALA191, LEU234, HIS230	TYR245, LEU232, VAL223, TYR248
	GLN227, HIS236, LEU243	PRO193, HIS236

While molecular docking provides initial insights into potential binding affinity and interaction modes from a static, structure-based perspective, docking scores and binding poses alone cannot fully capture protein conformational flexibility or the dynamic nature of ligand–receptor interactions. To address this limitation, MD simulations were subsequently employed to assess complex stability under near-physiological conditions by monitoring structural fluctuations, conformational adaptability, hydrogen bond persistence, and binding free energy over time.

Further, 500 ns MD simulations and MM-PBSA binding free energy calculations were crucial for distinguishing these candidates. The MD simulations revealed that pedalitin and salvianolic acid B exhibited partial ligand dissociation. Furthermore, free energy landscape analysis showed that the binding free energy of the MMP9–chlorogenic acid complex (−3.36 kJ/mol) was almost negligible, indicating unstable binding; thus, it was excluded from further consideration. In contrast, the MMP9–cirsiliol complex exhibited the most favorable binding free energy (ΔG_bind_ = −179.793 kJ/mol), consistent with the strong and persistent interactions observed in the MD simulations. These results indicate that cirsiliol is the most promising lead compound.

Beyond MMP9 inhibition, network pharmacology analysis indicates that cirsiliol may exert multi-target effects on other oncogenic signaling pathways in laryngeal cancer. The hub genes identified from the PPI network (such as EGFR, AKT1, and the MAPK family), which are involved in cancer growth, survival, EMT, and invasion, suggest that the anti-cancer function of cirsiliol may not be limited to MMP9 blockade but also involve the simultaneous regulation of other signaling cascades. Multi-target effects are common among many plant-derived natural products, such as flavonoids. While this may enhance therapeutic efficacy, it also carries the risk of side effects due to off-target interactions, which warrants further verification in mechanistic studies.

Cirsiliol is a naturally occurring polymethoxyflavonoid found in various medicinal plants, including R. Rubescens. It has gained increasing attention due to its broad pharmacological activities, including anti-inflammatory, antioxidant, and anticancer effects (Kang et al., 2013). Although previous studies have explored its mechanisms in other cancers, this study provides new insights demonstrating that cirsiliol is a potent and specific *in silico* binder of MMP9. This suggests a direct mechanism by which cirsiliol exerts anticancer effects in laryngeal cancer: by inhibiting key enzymes responsible for invasion and metastasis.

While our computational analyses provide a robust theoretical foundation, *in silico* predictions require experimental validation. Therefore, we further evaluated the cytotoxic and anti-migratory effects of cirsiliol in human cancer cell models. The compound exhibited clear dose-dependent growth inhibition in both TU177 and RK33 cells, with IC_50_ values of 31.59 μM and 17.73 μM, respectively. The lower IC_50_ in RK33 cells suggests cell type-dependent sensitivity. To investigate migration, TU177 cells were selected as a representative HNSCC model. The wound healing assay showed that treatment with 20 μM cirsiliol markedly delayed wound closure compared to the control, indicating effective inhibition of cell migration. Notably, knockdown of MMP9 in TU177 cells significantly suppressed migration, mirroring the effect observed with cirsiliol treatment. These findings suggest that cirsiliol inhibits cell migration at least in part by targeting MMP9, providing a mechanistic explanation for its anti-migratory activity. Together, these results demonstrate that cirsiliol not only reduces cell viability but also suppresses migratory behavior, highlighting its potential as a therapeutic agent for inhibiting tumor progression in head and neck cancer.

In summary, this study has expanded the potential therapeutic compound library for laryngeal cancer through a rigorous computational screening funnel. We not only provided structural insights into the interaction between plant-derived metabolites and MMP9 but also specifically identified cirsiliol as a highly promising MMP9 inhibitor. These *in silico* and *in vitro* findings provide strong theoretical and experimental support for the anticancer potential of cirsiliol, laying a solid foundation for the preclinical development of this natural compound for laryngeal cancer therapy.

## 5 Conclusion

In this study, through computational screening and experimental verification, we successfully characterized the anti-laryngeal cancer potential of *R*. *Rubescens*. Our network pharmacological analysis indicates that MMP9 is a key “executive” protein that drives ECM degradation and metastasis in head and neck squamous cell carcinoma. By leveraging high-throughput virtual screening and extended molecular dynamics simulations, we distinguished Cirsiliol from other candidate drugs (such as Pedalitin and chlorogenic acid) as a highly stable MMP9 inhibitor. *In vitro* biological experiments strongly confirmed the computational predictions. Millennium alcohol has obvious cytotoxicity to laryngeal cancer cell lines and can effectively inhibit cell migration. It is worth noting that the anti-migration effect of alizarol is similar to the phenotype observed in the MMP9 knockdown model, which provides strong evidence that its mechanism of action is at least partially mediated by MMP9 inhibition.

To further advance the development of *R*. *Rubescens* for head and neck tumor therapy, future studies will focus on conducting *in vivo* experiments and optimizing methods for enriching its bioactive constituents.

In conclusion, this study provides important structural and mechanistic insights into the interaction between herb-based metabolites and cancer targets. We have determined that *R*. *Rubescens* is a feasible therapeutic candidate. These research results provide a solid theoretical and experimental basis for the further development of *R*. *Rubescens* as a targeted therapeutic drug for inhibiting the invasion and metastasis of laryngeal cancer.

## Data Availability

The original contributions presented in the study are included in the article/supplementary material, further inquiries can be directed to the corresponding authors.
